# Polytetrafluoroethylene Felt Inlay Neomedia and Tissue Glue Do Not Prevent Reoperation in Type A Aortic Dissection

**DOI:** 10.3390/jcm13226663

**Published:** 2024-11-06

**Authors:** Jules Miazza, Luca Koechlin, Brigitta Gahl, Denis Berdajs, Luise Vöhringer, Friedrich Eckstein, Oliver Reuthebuch

**Affiliations:** 1 Department of Cardiac Surgery, University Hospital Basel, 4031 Basel, Switzerland; 2 Surgical Outcome Research Centre Basel, University Hospital Basel, University of Basel, 4056 Basel, Switzerland

**Keywords:** aortic dissection, Stanford type A, PTFE, French Glue, BioGlue, aortic root repair

## Abstract

**Background/Objectives:** Type A aortic dissection repair using Polytetrafluorethylene (PTFE) felt inlay and tissue glue has been proposed as a treatment modality. It remains unclear, if this method performs superiorly to tissue glue only. **Methods:** Between January 2011 and December 2015, 139 patients underwent surgical repair for type A aortic dissection, and 48 patients were excluded (*n* = 29 after receiving a composite graft, *n* = 18 in which no tissue glue was used, and *n* = 1 due to missing data). In the remaining patients, proximal aortic repair was performed either using PTFE felt inlay and tissue glue or tissue glue only. We analyzed the need for repeated surgery on the aorta during follow-up as a primary endpoint. The secondary endpoint was all-cause mortality at follow-up. Inverse probability of treatment weighting was used to balance the distribution of measured baseline covariates. **Results:** Sixty-six patients (73%) were treated with a tissue-glue-only approach—the Control Group. Twenty-five patients (27%) underwent proximal PTFE felt inlay and tissue glue—the Intervention Group. In the Intervention Group, 40% (*n* = 10) underwent reoperation due to re-dissection or pseudoaneurysm vs. 12% (*n* = 8) in the Control Group. The felt inlay increased the hazard of re-operation by 8.38 (1.63 to 43.0) after IPTW with death modeled as competing risk. **Conclusions:** Reoperation due to aortic complications was 10 times higher in patients treated with a combination of gluing and PTFE felt inlay vs. gluing only. These results are potentially caused by an interaction of PTFE, tissue glue, and aortic tissue.

## 1. Introduction

In the era of interventional cardiac therapies, the treatment of acute type A aortic dissection remains surgical [1,2]. However, type A aortic dissection is still associated with a considerable risk of mortality [3]. Surgical therapy of acute type A aortic dissection encompasses the replacement of the dissected ascending aortic segment. However, in cases where the dissection extends to the aortic root, aortic root replacement (composite graft procedure) or aortic root repair are surgical options.

In 2014, Rylski et al. reported promising results on a repair technique for the aortic root in type A aortic dissection. This technique consists of the reconstruction of the damaged aortic root portion by embedding a Polytetrafluorethylene (PTFE) felt inlay combined with tissue glue between the dissected aortic root layers. The underlying idea is an imitation of the media layer (Neomedia), causing fibrosis, thus improving stability and longevity [4]. The Neomedia technique was partly adopted at our institution following this publication. The aim of this study is the comparison of two different surgical treatments of acute type A dissection involving the aortic root: the use of tissue glue only (Control Group) vs. the use of PTFE felt inlay and tissue glue (Intervention Group).

## 2. Materials and Methods

### 2.1. Ethical Statement

This retrospective study was approved by the local ethical committee at the University of Basel, Basel, Switzerland on 3 January 2017. (Ethikkomission Nordwest und Zentralschweiz, EKNZ 2016-01953). Written informed consent was waived due to the study’s retrospective nature.

### 2.2. Patient Population

**Figure 1:**
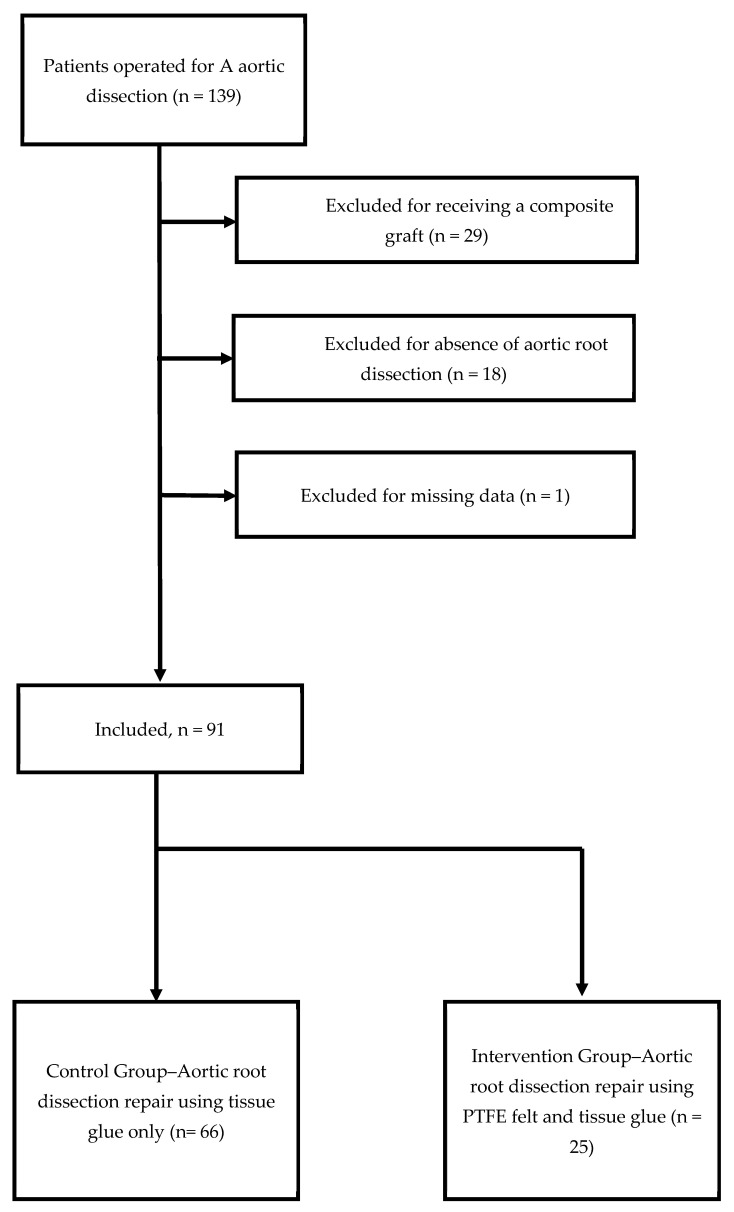
Patient selection.

In this retrospective, single-center analysis, all patients that received surgical therapy for acute type A aortic dissection at our institution from 1 January 2011 to 31 December 2015 were screened and *n* = 139 candidate were identified. To maintain a fair comparison of treatments, we excluded patients treated with a composite graft and patients in which no tissue glue was used due to the absence of aortic root dissection (*n* = 47). One patient was excluded due to missing data (Figure 1). Patients treated with PTFE felt inlay and tissue glue are referred to as the Intervention Group (IG). Patients treated with the standard approach (glueing of the dissected aortic root only) are referred to as the Control Group (CG). Patient selection is illustrated in Figure 1. Baseline characteristics, operative details, and postoperative complications were obtained from the prospectively maintained institutional registry (Intellect 1.7, Dendrite Clinical Systems, Henley-on-Thames, UK). Specific information on treatment details were extracted from patient records.

### 2.3. Follow-Up

As part of the clinical routine, patients who underwent aortic surgery were followed up according to an established protocol, including immediate post-operative computed tomography (CT) imaging and a first regular check-up six months after the index operation. Depending on the CT findings at first follow-up, further CT scans were planned to monitor the postoperative course and to determine the need for further treatment. Survival data were obtained from hospital records and collected in telephone interviews with patients’ general practitioners.

### 2.4. Outcomes

The primary outcome was the need for reoperation on the proximal aorta during follow-up. The secondary outcome was all-cause mortality during follow-up.

### 2.5. Operative Procedure

#### 2.5.1. Diagnostic

Type A aortic dissection was diagnosed using CT scan and/or transthoracic echocardiography (TTE). The diagnosis of type A aortic dissection was made according to the Stanford classification [5]. CT scan was used to examine aortic dimensions and to assess extent of perfusion impairment due to overlapping membranes and/or dissected vessels. Upon diagnostic confirmation, patients were transferred to the operating room.

#### 2.5.2. Surgical Technique

After sternotomy, cardiopulmonary bypass (CPB) was established. Arterial cannulation was achieved by cannulation of the subclavian artery or directly via the ascending aorta. Venous cannulation was achieved via the right atrium. In cases where the dissection membrane extended in the descending aorta, we opted, in most cases, for a thoracic stent (Conformable TAG Thoracic Endoprosthesis [CTAG]; W. L. Gore & Associates, Flagstaff, Ariz) implantation to stabilize the dissection membrane at the level of the distal aortic arch/descending aorta (Zone 3, [6,7]). In such cases, a J-wire was inserted from the non-dissected femoral artery to guide stent implantation. After initiation of CPB, the cross-clamped aorta was transected and cardioplegic arrest initiated via selective antegrade perfusion. After insertion of a left vent, hypothermia was initiated. At 28 °C bladder temperature, hypothermic circulatory arrest was established. Antegrade cerebral perfusion was assured using catheters (ACP, Distal Perfusion Catheters, LeMaitre Vascular Inc., Burlington, MA, USA) inserted into the left carotid artery, as well as into the brachiocephalic trunk. Blood temperature for cerebral perfusion was 20 °C with 300 to 500 mL/min flow. After resection of the diseased aortic tissue of the proximal aortic arch, a Gelweave Ante-Flo prosthesis (Vascuthek, Inchinnan Renfrewshire, Scotland, UK) was anastomosed to the distal aortic segment. CPB was then re-initiated through the prosthetic side arm and the situs meticulously de-aired. The sinus portion and the aortic valve were then inspected, and the dissected layers at the level of the proximal anastomosis to the prosthesis were either solely glued using tissue glue (Control Group—CG) or treated with a combination of PTFE felt inlay and tissue glue positioned between the dissection layers (Intervention Group—IG). In most cases, the aortic valve was reconstructed with re-suspension stitches at the commissures. For going off-bypass, patients were reperfused and rewarmed up to 36 °C bladder temperature.

#### 2.5.3. Types of Tissue Glue Used

The tissue glues used in both the intervention and the Control Group repair were BioGlue (CryoLife, Kennesaw, GA, USA) or French Glue (C.R. Bard Inc., Becton, Dickinson and Company, Franklin Lakes, NJ, USA). BioGlue, a two-component adhesive, consists of purified bovine serum albumin and glutaraldehyde. Tested in several laboratories, clinical and preclinical trials, its efficacy and safety have been proven and BioGlue is widely used in cardiac surgery [8,9]. French Glue is another surgical adhesive composed of formaldehyde, glutaraldehyde, gelatine, resorcinol, and calcium chloride. Due to its high concentration of formaldehyde, which may be toxic for cardiac tissue [10], French Glue is not approved by the U.S. Federal Drug Agency (FDA) for cardiac surgery. However, in Europe and Asia it has been used in cardiac surgery [11,12]. The efficacy of both tissue glues in type A aortic dissection repair has been proven and therefore the tissue glues are not primarily considered the source of the problem addressed in this study [11].

#### 2.5.4. Polytetrafluoroethylene(PTFE)-Felt

PTFE felt inlays are medical implants designed to add support to sutures in fragile tissues. In treatment of type A aortic dissection, a PTFE felt inlay is supposed to be an optimal material to stabilize the aortic wall by forming a Neomedia when embedded between the dissected layers [4]. In the literature, in a report of a reoperated patient, the PTFE felt inlay seemed to fuse with the native media, thus demonstrating potential stabilization capabilities [13].

### 2.6. Statistical Methods

The aim of this study was to analyze the difference in reoperation rate at follow-up between patients treated with tissue glue only (Control Group—CG) vs. PTFE felt inlay and tissue glue (Intervention Group—IG). Since type A aortic dissection is associated with high mortality, we consider it appropriate to take the risk of death into account in this analysis. We used inverse probability of treatment weighting (IPTW) to balance the distribution of measured baseline covariates between treatment groups, given the observational origin of the data. We included EuroSCORE 2, female sex, diabetes, dyslipidemia, family history, pulmonary disease, and NYHA as covariates in the propensity model. We stabilized the weights by replacing weights larger than 10 by 10. We calculated standardized differences for pre-operative variables before and after IPTW to assess residual imbalances between the groups. We used competing-risks regression before and after IPTW to calculate the sub-hazard ratio following the Fine and Grey approach [14] and treating death as competing event. After IPTW, we calculated robust standard errors. As a sensitivity analysis, we repeated the analysis after excluding all patients who died during the hospitalization. We assessed the proportional hazard assumption using Schoenfeld residuals. We then added type of glue as further covariate into the competing-risks regression to assess whether type of glue changes the risk of reoperation. All time-to-event analyses were carried out after right-censoring at 3 years of follow-up. Categorical variables were summarized as frequencies and percentages, and continuous variables were presented as mean ± standard deviation or as median with interquartile ranges, as appropriate. Comparisons between treatment groups were performed using Fishers exact test, *t*-test or Kruskal–Wallis rank test, as appropriate. Statistical analyses were performed by a biostatistician (BG) using Stata 16 (StataCorp, College Station, TX, USA).

## 3. Results

### 3.1. Baseline Characteristics

From January 2011 to December 2015, 139 patients underwent surgery for acute Stanford type A aortic dissection at our institution, and 29 (20%) patients were excluded from the analysis after having received a composite graft, 18 (13%) were excluded due to lack of aortic root dissection, and 1 patient (0.7%) was excluded due to missing data. Median (IQR) age was 64 (56 to 75) years, and 38% (*n* = 35) were female. The median [IQR] logistic EuroSCORE was 32% [21 to 51]. No statistical difference was observed in the baseline characteristics of the Control and Intervention Groups. Baseline characteristics are depicted in Table 1.

### 3.2. Operative Data

In 27% (*n* = 25) of patients, the proximal aorta was addressed using PTFE felt inlay and tissue glue (Intervention Group). In 73% (*n* = 66), the proximal aorta was treated using tissue glue only (Control Group). Operative, cardiopulmonary bypass, and aortic cross clamp time were comparable between the study groups. In both groups, most of the patients underwent a concomitant procedure. Deep hypothermic circulatory arrest (DHCA) was used in 96% of the Control Group and 100% of the Intervention Group (*p* = 1.00). Operative data are depicted in Table 2.

### 3.3. In-Hospital and Follow-Up Data

In-hospital mortality was 14% (*n* = 9) in the Control Group vs. 0% (*n* = 0) in the Intervention Group without statistically significant difference (*p* = 0.06). Median length of hospital stay was 13 days [8 to 21] and was comparable between treatment groups. Median follow-up time in the Control Group was 2.5 years [1.1 to 4.4] and 3.3 years [1.8 to 4.0 years] in the Intervention Group. In-hospital data are depicted in Table 3.

### 3.4. Reoperation at Follow-Up

During follow-up, mortality was comparable between both groups [Control Group 18% (*n* = 12) vs. Intervention Group 8% (*n* = 2), *p* = 0.33]. Overall mortality after follow-up, including in-hospital mortality, was also comparable between the groups [control 20% (*n* = 20) vs. intervention 20% (*n* = 5), *p* = 0.43], as depicted in Table 3. In the Intervention Group, a significantly higher incidence of reoperation due to repeated dissection was observed [control 12% (*n* = 8) vs. intervention 40% (*n* = 10), *p* = 0.006]. A calculation of standardized differences revealed differences of >0.1 between the Control Group and Intervention Group. To optimize group balancing, we performed IPTW, including EuroSCORE 2, female sex, diabetes, dyslipidemia, family history, pulmonary disease, and NYHA as covariates into the propensity model. However, standardized differences of pulmonary disease, creatinine clearance, smoking, and EuroSCORE 2 remained higher than 0.1 even after IPTW. Thus, we cannot exclude residual confounding. See Supplemental Table S1 for baseline characteristics with standardized differences before and after IPTW.

When accounting for time and the risk of death, the additional use of felt was associated with an increase in the risk of reoperation [sub-hazard ratio (SHR) 10.7 with confidence interval (CI) 95%, 2.28 to 50.2, *p* = 0.003 before IPTW and 8.38 with CI 1.63 to 43.0, *p* = 0.011 after IPTW]; see Table 4 for main results. When excluding all patients who died during the admission (*n* = 9) and treating death as competing event (sensitivity analysis), use of felt was associated with a 9.22-fold increase in the hazard of reoperation (CI 1.97 to 43.2, *p* = 0.005) before IPTW and 7.28 (CI 1.42 to 37.4, *p* = 0.017) after IPTW. We did not find an association of type of glue (BioGlue or FrenchGlue) and risk of reoperation (SHR 1.0002, CI 0.12 to 8.6, *p* = 1.0, SHR 1.36, CI 0.16 to 11.9, *p* = 0.78 after IPTW) when accounting for death. However, BioGlue was used only in eight patients in our study cohort, so these results might not be conclusive.

## 4. Discussion

In this retrospective, single-center study of patients undergoing surgery for acute type A aortic dissection, we investigated the impact of a surgical technique using a combination of PTFE felt inlay and tissue glue (Intervention Group) vs. tissue glue only (Control Group) for the repair of the dissected aortic root.

In 2014, Rylski et al. [4] reported their experience with the Neomedia technique using PTFE inlay and tissue glue in 489 patients. The authors reported an in-hospital mortality of 11% and a survival rate of 69 ± 2%, 50 ± 3% and 36 ± 5% at 5, 10 and 15 years. Furthermore, the authors reported a freedom from reoperation of 96 ± 1%, 92 ± 2% and 89 ± 4% over the same time period. However, in this study, no Control Group was available, precluding the possibility to draw definitive conclusions. In 2017, Tang et al. [15] provided further data on the performance of this technique with freedom of reoperation rate of up to 98 ± 2% at 10 years. Further studies comparing the use of this technique with tissue glue alone found no differences in survival or freedom from reoperation between both groups [16].

In our cohort, we found a 10-fold increased risk for re-operation in the Intervention Group vs. the Control Group. This result contrasts with the published literature, which subsequently will be discussed. First, the amount of tissue glue used during the procedure might interact with the profibrotic effect hypothesized by Rylski et al. [4]. Indeed, if the amount of tissue glue used is disproportionate, the aortic layer and the felt might not be in direct contact, thus impairing the profibrotic effect. Unfortunately, the exact amount of tissue glue used varies from patient to patient and is hard to monitor.

Second, even though tissue glue has been extensively used in the past for the treatment of type A aortic dissection involving the aortic root, a potential adverse effect in combination with PTFE and aortic tissue cannot be totally excluded. BioGlue, a bovine serum albumin/glutaraldehyde tissue glue, has been successfully used in cardiac surgery over the past years [9]. In a study from 2017, Ma et al. reported an incidence of 0.6% for anastomotic pseudoaneurysm formation in a study cohort of 233 patients who underwent aortic root reconstruction for aortic aneurysm, aortic dissection, intramural hematoma, pseudoaneurysm, coarctation, or penetrating aortic ulcer [17]. However, in this study, PTFE felt was only applied on the external edge of the dissected aortic root, thus providing insights on the gluing ability of BioGlue but not on its interaction with PFTE. Moreover, according to the manufacturer, BioGlue is not specifically designed for a concomitant use with PFTE. This combination is, however, not listed as a contraindication. On request, the company declared not to be aware of any adverse interaction of BioGlue with PTFE.

Third, the other type of tissue glue used in our study, French Glue, has been used as an adhesive in cardiac surgery for decades; its application is well established in Europe and Asia and is considered effective [11,18]. However, in the USA, the FDA has not approved French Glue. The toxic effects of residual formaldehyde on tissue after chemical reaction have raised controversy and no concluding guidelines have been defined to date. In a study from 2001, Kazui et al. [18] found possible histologic signs for tissue necrosis (disappearance of smooth muscle cell nuclei) at the site of French Glue application in a patient reoperated after type A aortic dissection treated with French Glue. On the other hand, in 2012, Hata et al. [12] reported on the 3-year outcome of 25 patients who underwent surgery for type A aortic dissection and in whom FrenchGlue was applied and did not observe any dilatation or re-dissection during follow-up in this subgroup of the study cohort. In this study, however, no patient was reoperated, precluding from drawing histological conclusions. When contacted, the manufacturer referred to the published literature [11,12,18].

Fourth, even though the reoperation rate was high in the Intervention Group, this did not affect mortality at follow-up. This highlights the fact that, when performed at experienced centers, reoperative surgery carries an acceptable risk burden.

## 5. Conclusions

In this study, the use of a technique combining PTFE felt inlay in combination with tissue glue for the repair of the dissected aortic root in type A aortic dissection did not provide any benefit compared to tissue glue only and was even associated with a higher risk of reoperation at follow-up.

## Figures and Tables

**Table 1:** Baseline characteristics.

	Total (*n* = 91)	Control Group (*n* = 66)	Intervention Group (*n* = 25)	*p*
Age, y	64 [56 to 75]	65 [56 to 76]	64 [58 to 70]	0.48
Female	35 (38%)	29 (44%)	6 (24%)	0.10
BMI, kg/m^2^	26 [24 to 31]	25 [23 to 30]	27 [25 to 31]	0.35
Diabetes mellitus	12 (13%)	11 (17%)	1 (4.0%)	0.17
Dyslipidemia	22 (24%)	19 (29%)	3 (12%)	0.11
Arterial hypertension	55 (60%)	41 (62%)	14 (56%)	0.64
Smoker	33 (36%)	23 (35%)	10 (40%)	0.81
Positive cardiovascular family history	14 (15%)	12 (18%)	2 (8.0%)	0.33
Pulmonary disease	14 (15%)	12 (18%)	2 (8.0%)	0.33
Renal disease	5 (5.5%)	5 (7.6%)	0 (0.00%)	0.32
Creatinine clearance, ml/min	79 [60 to 108]	78 [55 to 108]	81 [69 to 111]	0.24
Coagulopathy	43 (47%)	31 (47%)	12 (48%)	1.00
Previous cerebrovascular event	32 (35%)	23 (35%)	9 (36%)	1.00
Angina CCS III/IV	16 (18%)	14 (21%)	2 (8.0%)	0.22
NYHA III/IV	22 (24%)	15 (23%)	7 (28%)	0.59
Ejection fraction, %	60 [55 to 60]	60 [55 to 60]	60 [60 to 60]	0.76
Logistic EuroSCORE, %	32 [21 to 51]	33 [22 to 51]	29 [20 to 45]	0.61
EuroSCORE 2, %	13 [7.1 to 23]	13 [7.0 to 26]	11 [7.2 to 22]	0.66

Data presented as number and percentage and median and interquartile range according to the distribution. BMI Body Mass Index, CCS Canadian Cardiovascular Society, NYHA New York Heart Association.

**Table 2:** Operative data.

	Total (*n* = 91)	Control Group (*n* = 66)	Intervention Group (*n* = 25)	*p*
Emergency/Salvage	87 (96%)	62 (94%)	25 (100%)	0.57
Concomitant procedure				
CABG	11 (12%)	10 (15%)	1 (4.0%)	0.28
Aortic valve repair	52 (57%)	34 (52%)	18 (72%)	0.10
Aortic valve replacement	10 (11%)	7 (11%)	3 (12%)	1.00
Mitral valve repair	0 (0%)			
Operation time, min	210 [195 to 270]	240 [200 to 270]	210 [180 to 240]	0.06
CPB time, min	128 [109 to 154]	129 [109 to 163]	128 [107 to 150]	0.68
Aortic cross clamp time, min	76 [65 to 85]	75 [64 to 83]	78 [71 to 89]	0.23
Deep hypothermic cardiac arrest	90 (99%)	65 (98%)	25 (100%)	1.00
Red blood cell transfusion needed	60 (66%)	45 (68%)	15 (60%)	0.47
Proximal PTFE inlay felt and tissue glue	25 (27%)	0 (0.00%)	25 (100%)	<0.001
BioGlue *	8 (8.8%)	3 (4.5%)	5 (20%)	0.033
French Glue *	82 (90%)	62 (94%)	20 (80%)	0.11

Data presented as number and percentage and median and interquartile range according to the distribution. CABG Coronary Artery Bypass Grafting, CPB Cardiopulmonary Bypass, PTFE Polytetrafluoroethylene. * note that in one patient in the Control Group, type of glue was not documented.

**Table 3:** In-hospital and follow-up data.

	Total (*n* = 91)	No Felt Used (*n* = 66)	Use of Felt and Glue (*n* = 25)	*p*
Re-operation for bleeding	4 (4.4%)	3 (4.5%)	1 (4.0%)	1.00
Length of hospital stay, days	13 [8.0 to 21]	12 [8.0 to 20]	14 [8.0 to 22]	0.58
In-hospital mortality	9 (10%)	9 (14%)	0 (0.00%)	0.06
Follow-up, years	2.7 [1.4 to 4.3]	2.5 [1.1 to 4.4]	3.3 [1.8 to 4.0]	0.39
Reoperation within 3 years	4 (4.4%)	2 (3.0%)	2 (8.0%)	0.30
Mortality within 3 years	14 (15%)	12 (18%)	2 (8.0%)	0.33
Reoperation during follow-up	18 (20%)	8 (12%)	10 (40%)	0.006
Reasons for reoperation				
Aortic insufficiency	12 (13%)	6 (9%)	6 (24%)	0.083
Aortic aneurysm	10 (11%)	5 (8%)	5 (20%)	0.13
Overall mortality	25 (27%)	20 (30%)	5 (20%)	0.43
Cause of mortality				
Cardiac	10 (11%)	9 (14%)	1 (4%)	0.27
Infectious	3 (3%)	2 (3.0%)	1 (4%)	1.00
Respiratory	1 (1%)	1 (2%)	0 (0%)	1.00
Neurologic	2 (2%)	2 (3.0%)	0 (0%)	1.00
Mesenteric ischemia	1 (1%)	1 (2%)	0 (0%)	1.00
Unknown	8 (9%)	5 (8%)	3 (12%)	0.68

Data presented as number and percentage and median and interquartile range according to the distribution.

**Table 4:** Association of use of felt with risk of reoperation, death modeled as competing risk.

	Before IPTW		After IPTW	
	Hazard Ratio (95% CI)	*p*	Hazard Ratio (95% CI)	*p*
Main analysis: Entire cohort				
Re-operation	10.7 (2.28 to 50.2)	0.003	8.38 (1.63 to 43.0)	0.011
Death without re-operation	0.47 (0.10 to 2.11)	0.323	0.43 (0.10 to 1.93)	0.270
Death	0.40 (0.09 to 1.80)	0.234	0.36 (0.08 to 1.58)	0.176
Sensitivity analysis *				
Re-operation	9.22 (1.97 to 43.2)	0.005	7.28 (1.42 to 37.4)	0.017
Death without re-operation	2.48 (0.35 to 17.6)	0.363	1.98 (0.27 to 14.4)	0.499
Death	1.51 (0.25 to 9.04)	0.652	1.19 (0.20 to 7.02)	0.849

* patients who died in-hospital were excluded. Note that estimates represent sub-hazard ratios for re-operation and death without re-operation, whereas usual hazard ratios are shown for death.

## Data Availability

Data will be shared upon reasonable request.

## Supplementary Materials

The following supporting information can be downloaded at: https://www.mdpi.com/article/10.3390/jcm13226663/s1, Table S1: baseline characteristic before and after IPTW.
